# Supplementary material for: Understanding of arthrofibrosis: New explorative insights into extracellular matrix remodeling of synovial fibroblasts
Source: PLoS One. 2023 May 26;18(5):e0286334. doi: 10.1371/journal.pone.0286334 (PMC10218749; doi:10.1371/journal.pone.0286334)
Supplement: S1 Table — (PDF) [file pone.0286334.s001.pdf]

Supplementary Materials

S1 Table. Primer sequences and annealing temperatures (T_A) used for qRT-PCR analysis.

Gene	Primer sequence 5'-3'	T_A / °C	Product Size / bp
ACAN	CACCCCATGCAATTTGAG<br>GCCACTGTGCCCTTTTTA	59	158
BGN	AGGACCTGCTTCGCTACT<br>GGGCACGGGGTTGTTG	63	290
B2M	TGTGCTCGCGCTACTCTCTCTT<br>CGGATGGATGAAACCCAGACA	59	137
COL1A1	GATGTGCCACTCTGACT<br>GGGTTCTTGCTGATG	63	151
COL3A1	AGTCTGGAGTAGCAGTAG<br>AGCTGGACCTTTGATACC	63	281
DCN	CCTTCCGCTGTCAATG<br>GCAGGTCTAGCAGAGTTG	63	102
GAPDH	AGGTCGGAGTCAACGGAT<br>TCCTGGAAGATGGTGATG	59	223
HPRT1	GCTGACCTGCTGGATTAC<br>TGCGACCTTGACCATCTT	59	258
HSPG2	TGAACCCACAGCGAAAC<br>GTGTAGGAGAGGGTGTATC	59	190
SDC2	GGAGCTGATGAGGATGTA<br>AATGACAGCTGCTAGGAC	59	279
VCAN	CAAGACACGGTGTCACTG<br>AAGAGCTGCTCTGGAGTT	59	123
XYLT1	GAAGCCGTGGTGAATCAG<br>CGGTCAGCAAGGAAGTAG	63	281
XYLT2	ACACAGATGACCCGCTTGTGG<br>TTGGTGACCCGCAGGTTGTTG	63	139
