# Supplementary material for: Understanding of arthrofibrosis: New explorative insights into extracellular matrix remodeling of synovial fibroblasts
Source: PLoS One. 2023 May 26;18(5):e0286334. doi: 10.1371/journal.pone.0286334 (PMC10218749; doi:10.1371/journal.pone.0286334)
Supplement: S1 Fig — (PDF) [file pone.0286334.s002.pdf]

Supplementary Materials

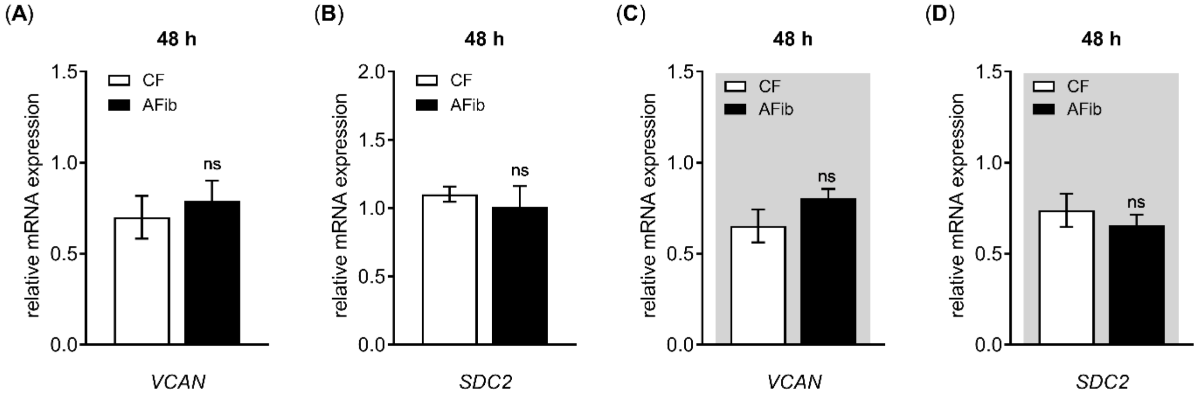

S1 Fig. No differences in basal and TGF-β1-induced *VCAN* and *SDC2* mRNA expression in AFib and CF. Human primary AFib (n = 2) and CF (n = 2) were cultured the day before the experiment. Cells were serum-starved for 24 h and maintained in (A,B) serum-reduced or (C,D) TGF-β1 (5 µg/L, gray shaded) supplemented media for an additional 48 h. Relative expression levels of the PG genes (A,C) *VCAN* and (B,D) *SDC2* were analyzed by qRT-PCR. Data shown are means ± SEM for three biological and three technical replicates per experiment. Mann-Whitney *U* test: not significant (ns).
