# Supplementary material for: Understanding of arthrofibrosis: New explorative insights into extracellular matrix remodeling of synovial fibroblasts
Source: PLoS One. 2023 May 26;18(5):e0286334. doi: 10.1371/journal.pone.0286334 (PMC10218749; doi:10.1371/journal.pone.0286334)
Supplement: Attachment — Submitted filename: Rebuttal Letter.pdf [file pone.0286334.s003.pdf]

Dominik R. Haudenschild, Ph. D.

Academic Editor

PLOS ONE

Rebuttal Letter

Manuscript ID: PONE-D-22-27716

February 24, 2023

Dear Dr. Haudenschild,

We would like to thank you for your email dated 10th of January 2023 enclosing the reviewers' comments and the opportunity to revise our manuscript. We also appreciate the reviewers for their feedback and revised the latest manuscript version which you can find attached. All changes made to the latest manuscript version are shown via Microsoft Word "Track Changes" function. We have copied the reviewers' comments (black) from the submission menu of the editorial manager to provide a point-by-point response (red) to the comments raised. The revision has been made in consultation with all co-authors. We hope that the revised manuscript is now suitable for publication in PLOS ONE.

We thank you for your time and look forward to hearing from you.

Yours sincerely,

Isabel Faust-Hinse

Dr. Isabel Faust-Hinse

Herz- und Diabeteszentrum NRW

Institut für Laboratoriums- und Transfusionsmedizin

Georgstraße 11, 32545 Bad Oeynhausen, Germany

Phone: (+49) 5731/97-1234; Fax: (+49) 5731/97-2352

Email: ifaust-hinse@hdz-nrw.de

Reviewer 1

This manuscript describes the remodeling of the extracellular matrix of synovial primary fibroblasts obtained from arthrofibrotic tissues. The study was performed using standard methods on cellular and molecular biology and statistical analysis was used correctly. Limitations of this study are presented in the Discussion and the Conclusions. Indeed, this study opens new issues for a more detailed examination of the kind of GAGs increased after TGF-beta treatment rather than the whole amount of GAGs, the activity of the altered MMPs found in PCR array etc.

We thank the reviewer for this positive feedback.

Reviewer 2

The manuscript by Ly et al investigated the differences in cultured fibroblasts isolated from healthy individuals and patients with arthrofibrosis, in terms of xylosyltransferase-1 activity important for collagen crosslinking, collagen and proteoglycan expression, fibrotic genes expression, and cellular contractility, in the presence or absence of TGFb. The manuscript is well-written in a manner that is clear and concise. The major concern, as the authors pointed out, was the lack of sufficient biological replicates and this significantly reduced the scientific rigor of this study and could potentially alter the study's major conclusion. Although the authors have identified novel genes potentially involved in regulation of arthrofibrosis, the functions and roles of these genes in fibrosis should have been investigated further in this study, as many of the results from this study are not novel and have been published in other areas of fibrosis.

We thank the reviewer for his or her feedback. Because chronic fibrosis affects multiple organ systems, one potential strategy for treating fibrosis is to target common mechanisms and central signaling pathways that are of key pathophysiological importance in various fibrotic diseases. The molecular and cellular processes underlying fibrotic diseases share many functional similarities despite differences in etiology and clinical outcome. Because of the common fibrosis mechanisms in many diseases, research performed on one human organ system can be translated to other tissues. Therefore, it is very important that all researchers working on human fibrosis share their knowledge of fibrosis biology with the scientific community.

Major points: 1. In Fig 2, based on the XT activity assays, the authors concluded that there was an increase in XT expression or secretion at 96h. However, this could be due to an increase in the enzymatic activity rather than an increase in enzyme expression or secretion, XYLT1 mRNA level should be determined at 96 h.

We would like to thank the reviewer for the constructive feedback and want to explain our reason for waiving the XYLT1 mRNA expression determination at the 96-h time point and just focusing on the 2-h time point. As shown in previous works there is a good correlation between the mRNA expression level and the extracellular XT-I activity of the cell culture system, irrespective of the cell type used (see references below).

Faust I, Roch C, Kuhn J, Prante C, Knabbe C, Hendig D. Human xylosyltransferase-I - a new marker for myofibroblast differentiation in skin fibrosis. Biochem Biophys Res Commun. 2013;436(3):449-454. doi:10.1016/j.bbrc.2013.05.125

Prante C, Milting H, Kassner A, et al. Transforming growth factor beta1-regulated xylosyltransferase I activity in human cardiac fibroblasts and its impact for myocardial remodeling. J Biol Chem. 2007;282(36):26441-26449. doi:10.1074/jbc.M702299200

Ly T-D, Wolny M, Lindenkamp C, Birschmann I, Hendig D, Knabbe C, Faust-Hinse I. The Human Myofibroblast Marker Xylosyltransferase-I: A New Indicator for Macrophage Polarization. Biomedicines. 2022; 10(11):2869. https://doi.org/10.3390/biomedicines10112869

This information can also be found in the main text in lines 548 f. "It was shown that an increase in extracellular XT activity correlates with a former XYLT1 mRNA increase [9,30,35]."

In addition, quantification of extracellular XT-I activity from the cell culture supernatant is based on the enzyme-catalysed incorporation of [14C]-D-xylose from UDP-[14C]-D-xylose into silk fibroin as the XT acceptor substrate, as indicated in lines 260 f. Therefore, an increase in enzymatic activity in this cell-free assay approach is due to a higher amount of enzyme in the sample (cell culture supernatant) and

not to increased enzyme activity of the cell itself. Furthermore, to the best of our current knowledge, there is no known regulation of enzyme activity due to structural changes (see reference below) or translational modifications that would lead to increased XT activities.

Briggs DC, Hohenester E. Structural Basis for the Initiation of Glycosaminoglycan Biosynthesis by Human Xylosyltransferase 1. Structure. 2018;26(6):801-809.e3. doi:10.1016/j.str.2018.03.014

It is known from previous works that cytokine-mediated XYLT1 mRNA expression increases lead to time-dependent intracellular protein expression increases that decline over time to a constant baseline level as the constitutive active XT-I protein is secreted into the extracellular space (see references below).

Khair M, Bourhim M, Barré L, et al. Regulation of xylosyltransferase I gene expression by interleukin 1β in human primary chondrocyte cells: mechanism and impact on proteoglycan synthesis. J Biol Chem. 2013;288(3):1774-1784. doi:10.1074/jbc.M112.419887.

Ly TD, Plümers R, Fischer B, et al. Activin A-Mediated Regulation of XT-I in Human Skin Fibroblasts. Biomolecules. 2020;10(4):609. Published 2020 Apr 14. doi:10.3390/biom10040609

Therefore, quantification of secreted XT-I protein from cell culture supernatants provides information about previous intracellular protein increases that were due to former XYLT1 mRNA expression changes. Consequently, quantification of an mRNA level at a particular time point, such as the proposed 96 h time point, does not necessarily correlate with the protein level at the same time point.

Also, while the author did investigate the basal XYLT1 mRNA level at 2 h and claimed that this time frame is sufficiently short to exclude any residue cytokine effects and epigenetic changes carried over from previous in vivo conditions, they did not cite any supporting reference. Additional experiments aim to detect epigenetic differences in these cells should be included.

We thank the reviewer for this comment. We agree with him or her that the fundamental basis of all cell culture studies performed with primary cells is the phenotype of the patient-derived primary cells, as they retain their in vivo phenotype when they are grown in vitro as a result of epigenetics (lines 112 ff., lines 135 f.).The temporally XT activity increase observed in AFib may result from higher cell density, increased cytokine concentrations or epigenetic changes (methylation, histone modifications or non-coding RNAs) from the former ***in vivo*** *profibrotic milieu leading to XYLT1 mRNA expression changes. In brief, as written in line 339 f., "the temporary XT activity increase (..) may result from former XYLT1 (..) expression changes that were introduced* ***in vivo*** *and retained upon* ***in vitro*** *culture (..)" due to epigenetics. In addition, the expression of other factors such as surface receptors, transcription factors, cytokines etc. can also be changed, thus contributing to the activated fibroblast phenotype observed in AFib. As cell density was already taken into account when determining XT activity (assay normalization), it was assumed that the higher XT activity observed in AFib was due to autocrine effects of secreted cytokines or to the presence of epigenetics changing basal XYLT1 mRNA expression. In previous studies, we have shown that epigenetic changes, such as altered microRNA levels, can affect the "basal"/steady-state XYLT1 mRNA expression in the context of fibrosis. To determine basal XYLT1 mRNA expression levels of control and patient-derived primary cells, serum deprivation was performed for 24 hours, followed by media change and determination of mRNA expression after 3 h. This determination of mRNA expression is based on previous studies by our group showing that cytokine-mediated XYLT1 expression changes occur at high cytokine concentrations at 3 h, whereas physiological cytokine concentrations produced by the cells themselves were not sufficient to have visible effects on the relative mRNA expression determined at 3 hours. Therefore the 2-h time point chosen in the present study is sufficient to rule out autocrine cytokine stimulation on basal XYLT1 expression of AFib (mentioned in lines 341 ff.). Nevertheless, we agree with the reviewer that the supporting references (see below) to the chosen experimental design were missing and added the information on page 16, line 342. The chosen culture duration of 2 hours was selected in analogy to previous work, in order to exclude any in vitro cytokine-mediated effect on the detected expression changes.*

Faust I, Roch C, Kuhn J, Prante C, Knabbe C, Hendig D. Human xylosyltransferase-I - a new marker for myofibroblast differentiation in skin fibrosis. Biochem Biophys Res Commun. 2013;436(3):449-454. doi:10.1016/j.bbrc.2013.05.125

Ly TD, Plümers R, Fischer B, et al. Activin A-Mediated Regulation of XT-I in Human Skin Fibroblasts. Biomolecules. 2020;10(4):609. Published 2020 Apr 14. doi:10.3390/biom10040609

Ly, T.-D.; Kleine, A.; Plümers, R.; Fischer, B.; Schmidt, V.; Hendig, D. Cytokine-Mediated Induction of Human Xylosyltransferase-I in Systemic Sclerosis Skin Fibroblasts. Biochemical and Biophysical Research Communications 2021, 6.

Ly, T.-D.; Riedel, L.; Fischer, B.; Schmidt, V.; Hendig, D.; Distler, J.; Kuhn, J.; Knabbe, C.; Faust, I. MicroRNA-145 Mediates Xylosyltransferase-I Induction in Myofibroblasts via Suppression of Transcription Factor KLF4. Biochemical and Biophysical Research Communications 2020, 523, 1001–1006, doi:10.1016/j.bbrc.2019.12.120.

Furthermore, we agree with the reviewer that it would be highly interesting to perform epigenetic studies on the cells used here. Unfortunately, the scope of such studies, both in terms of time and content, would be beyond the scope of this manuscript.

2. While there were increased expression in collagens and proteoglycans in fibrotic vs control cells as expected, the results in the presence of TGF were confounding as the study showed that TGF actually inhibited the levels of collagens and proteoglycans in fibrotic cells. This is contradictory to the known effects of TGF as a profibrotic agents. What is the authors' explanation on this?

We thank the reviewer for this feedback and think that there is a misunderstanding regarding the data normalization. All normalized gene expression levels were referred to the respective target gene expression of one control fibroblast sample for relative comparison of multiple biological samples per experiment. This means in the case of Figure 4C that the relative gene expression level was normalized to one of the two used control fibroblast primary cultures (3 biological replicates) that was also treated with TGF-β1. Thus, it is not possible to directly compare values between two graphs of Figure 4, but rather within a graph. Below you can find the same data (Figure 4A-D) normalized to one of the two used control fibroblast primary cultures that was not treated with TGF-β1. As you can see, the TGF-β1 treatment of CF as well as AFib resulted in a relative increase compared to the respective untreated controls. Since this resembles no new information (Chen, S.-J.; Yuan, W.; Mori, Y.; Levenson, A.; Varga, J.; Trojanowska, M. Stimulation of Type I Collagen Transcription in Human Skin Fibroblasts by TGF-β: Involvement of Smad 3. Journal of Investigative Dermatology 1999, 112, 49–57), we decided to directly compare CF and AFib that were treated either with or without TGF-β1. The same was done for the PG expression data. TGF-β1 treatment of SF led to diminished ACAN and DCN mRNA expression, while TGF-β1 treatment increased the expression of BGN and HSPG2 (see examples below). The same effects were observed for AFib, explaining our decision to directly compare the CF and AFib that were treated either with or without TGF-β1 as well. The information about the data normalization can be found in the figure captions (Figure 4 and 6): "The relative gene expression values were related to the respective CF treatments without or with TGF-β1".

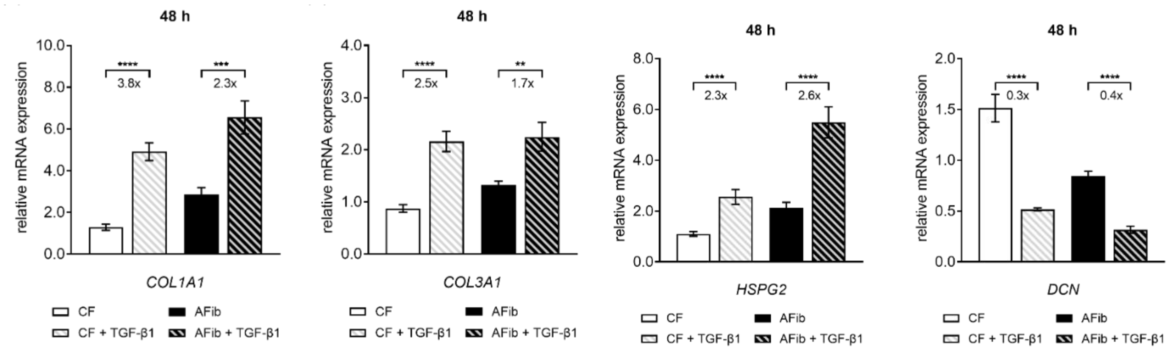

The dosage of TGF has been shown to be important in determining its biological effects, as too high a dose could have an opposite effect. Due to manufacturer and batch variations in TGFb, have the authors done a titration experiment to determine the optimal dosage for the TGF used in their studies?

We thank the reviewer for this feedback. Based on the literature (see references below), the TGF-β1 concentration used in primary synovial fibroblast cultures is usually 10 ng/mL. As demonstrated in the figures above, the TGF-β1 concentration of 5 ng/mL was ideal to achieve an apparent gene expression induction in CF and AFib. At this concentration, cells were highly proliferative and showed no signs of apoptosis induction by microscopic analysis (Fig. 3 and 5). Thus, we decided to perform our experiments with 5 ng/mL instead of 10 ng/mL TGF-β1 to avoid the indicated opposite effects.

Cheon, H.; Yu, S.-J.; Yoo, D.H.; Chae, I.J.; Song, G.G.; Sohn, J. Increased Expression of Pro-Inflammatory Cytokines and Metalloproteinase-1 by TGF- β 1 in Synovial Fibroblasts from Rheumatoid Arthritis and Normal Individuals. Clinical and Experimental Immunology 2002, 127, 547–552, doi:10.1046/j.1365-2249.2002.01785.x.

Ruscitti, P.; Liakouli, V.; Panzera, N.; Angelucci, A.; Berardicurti, O.; Di Nino, E.; Navarini, L.; Vomero, M.; Ursini, F.; Mauro, D.; et al. Tofacitinib May Inhibit Myofibroblast Differentiation from Rheumatoid-Fibroblast-like Synoviocytes Induced by TGF-β and IL-6. Pharmaceuticals 2022, 15, 622, doi:10.3390/ph15050622.

Sakuma, M.; Hatsushika, K.; Koyama, K.; Katoh, R.; Ando, T.; Watanabe, Y.; Wako, M.; Kanzaki, M.; Takano, S.; Sugiyama, H.; et al. TGF- Type I Receptor Kinase Inhibitor down-Regulates Rheumatoid Synoviocytes and Prevents the Arthritis Induced by Type II Collagen Antibody. International Immunology 2006, 19, 117–126, doi:10.1093/intimm/dxl128.

Minor points:
1. There is a typo in line 285, “Aecretion”

We thank the reviewer for this remark and corrected the word (line 291).

2. In line 354, “biological replicates” should be technical replicates.

We thank the reviewer for this comment and would like to explain to him or her why we chose the term "biological replicates" here. All our experiments were performed with the following level of replication:

- *n corresponds to the number of independent donor-derived primary cell cultures*
- *"Biological replicate" refers to the number of cell culture replicates (number of independent wells) created per donor*
- *"Technical replicate" refers to the number of measurements performed per cell culture replicate*

Therefore, biological variability is much greater than technical variability, so it is favourable to apply resources to sampling biologically relevant variables unless the technical variability measurements are also of interest, in which case we have reported the number of measurements (technical replicates) performed per biological replicate. If only one technical measurement was performed per biological replicate, it is incorrect to speak of "technical replicates", therefore only the number of biological replicates is indicated in the figure caption.
